# Immunodominance of Epitopes and Protective Efficacy of HI Antigen Are Differentially Altered Using Different Adjuvants in a Mouse Model of *Staphylococcus aureus* Bacteremia

**DOI:** 10.3389/fimmu.2021.684823

**Published:** 2021-05-27

**Authors:** Zhifu Chen, Qiang Gou, Qingshan Xiong, Lianli Duan, Yue Yuan, Jiang Zhu, Jintao Zou, Longlong Chen, Haiming Jing, Xiaoli Zhang, Ping Luo, Hao Zeng, Quanming Zou, Zhuo Zhao, Jinyong Zhang

**Affiliations:** ^1^ National Engineering Research Center of Immunological Products, Department of Microbiology and Biochemical Pharmacy, College of Pharmacy, Third Military Medical University, Chongqing, China; ^2^ Department of Pathology, Southwest Hospital, Third Military Medical University, Chongqing, China; ^3^ Department of Clinical Hematology, College of Pharmacy, Third Military Medical University, Chongqing, China

**Keywords:** *Staphylococcus aureus*, immunodominant epitope, antibody, adjuvants, bacteremia

## Abstract

HI, a fusion protein that consists of the alpha-toxin (Hla) and the N2 domain of iron surface determinant B (IsdB), is one of the antigens in the previously reported *S. aureus* vaccine rFSAV and has already entered phase II clinical trials. Previous studies revealed that HI is highly immunogenic in both mice and healthy volunteers, and the humoral immune response plays key roles in HI-mediated protection. In this study, we further investigated the protective efficacy of immunization with HI plus four different adjuvants in a mouse bacteremia model. Results showed that HI-mediated protection was altered in response to different adjuvants. Using antisera from immunized mice, we identified seven B-cell immunodominant epitopes on Hla and IsdB, including 6 novel epitopes (Hla_1-18_, Hla_84-101_, Hla_186-203_, IsdB_342-359_, IsdB_366-383_, and IsdB_384-401_). The immunodominance of B-cell epitopes, total IgG titers and the levels of IFN-γ and IL-17A from mice immunized with HI plus different adjuvants were different from each other, which may explain the difference in protective immunity observed in each immunized group. Thus, our results indicate that adjuvants largely affected the immunodominance of epitopes and the protective efficacy of HI, which may guide further adjuvant screening for vaccine development and optimization.

## Introduction

The World Health Organization (WHO) released a list of ‘priority pathogens’ in 2007, which contains 12 drug-resistant bacteria that pose a great threat to public health and for which alternative therapeutic strategies are urgently required (1). *Staphylococcus aureus* (*S. aureus*) ranks first among all Gram-positive bacteria among these drug-resistant pathogens and is involved in a variety of severe infections with high morbidity and mortality (2, 3). Several vaccine candidates against this bacterium have been developed, and some of them have entered clinical trials over the past two decades (4–7). However, none of these vaccines has yet been approved for clinical use (8).

rFSAV is a recombinant five-antigen *S. aureus* vaccine developed by our lab and is now under evaluation in a phase II clinical trial (CTR20160004, http://www.chinadrugtrials.org.cn/). HI, one of the antigens within rFSAV, is a fusion protein consisting of the H35L mutant of alpha-hemolysin (Hla) and the iron surface determinant B N2 domain (IsdB-N2) (9). Previous studies revealed that immunization with HI alone provides partial protection against *S. aureus* infection (10), and passive immunization with HI-specific polyclonal antibodies showed protection in a murine sepsis model, indicating that the humoral immune response is essential in HI-mediated protection (11). Since a few immunodominant epitopes in an antigen are involved in inducing a robust response rather than the other epitopes, the specificity and response of the immunodominant epitopes can easily be determined (12). Taking this into consideration, we identified 3 immunodominant epitopes on HI using serum from immunized individuals in a phase I clinical trial, which was performed to evaluate the safety and immunogenicity of rFSAV. Monoclonal antibodies recognizing these epitopes were also protective against *S. aureus* infection (13), further confirming the importance of immunodominant epitopes for inducing protective immunity.

An adjuvant is defined as a formulation that enhances the level of immunogenicity or changes the type of immune response toward a given antigen. To date, several adjuvants have been approved for clinical administration, such as the classical alum adjuvant MF59 and the AS series (14). As the mechanism of these adjuvants varies greatly, the addition of different adjuvants may impact the immunodominant epitope spectrum of an antigen, which may further affect the protective efficacy of a vaccine (15). Furthermore, rFSAV contains HI and three other antigens, and the immune responses induced by the other antigens in rFSAV might impact the response to HI; thus, it is necessary to conduct epitope mapping using serum from individuals immunized with HI alone.

To date, selection of an adjuvant is based on its ability to enhance the immunogenicity and efficacy of the vaccine (16). Knowledge of how adjuvants modulate the specificity of the antibody response against B epitopes would be of great interest for choosing adjuvants in a more rational way. In the present study, to gain further insight into the protective efficacy of HI-induced immunity, we immunized mice with HI formulated with AlPO_4_, complete Freund’s adjuvant (CFA), AddaVax and c-di-AMP. The antisera from each immunized mouse were used for epitope mapping. Herein, we report the difference in protective efficacy and immunodominant epitopes identified from these preparations.

## Materials and Methods

### Ethics Statement

All animal experiments performed in this study were approved by the Animal Ethical and Experimental Committee of the Third Military Medical University (Chongqing, Permit No. 2011-04) in accordance with their rules and regulations.

### Animal and Antigens

Six- to eight-week-old specific pathogen-free female BALB/c mice were purchased from Beijing HFK Bioscience Co., Ltd. (Beijing, China). The HI antigen was expressed and purified as previously described (10).

### Peptide Synthesis and KLH Conjugations

Eighteen-mer peptides with 12 amino acid length overlaps to cover the full lengths of Hla (Sequence ID: ADQ77533.1) and IsdB-N2 (Sequence ID: WP_031875332.1) were synthesized and purified by China Peptides Co., Ltd. OVA_192–201_ (EDTQAMPFRV) was used as a negative control peptide. The purity of these peptides was expected to be 95% or higher. The peptides were dissolved in dimethyl sulfoxide at a concentration of 0.5 mg/mL and stored at -80°C before use. For every immunodominant peptide, peptide keyhole limpet hemocyanin (KLH) conjugations were performed by China Peptides Co., Ltd.

### Immunization and Infection

To determine the protective efficacies of HI, mice were randomized into different groups (n=10) and intramuscularly immunized with 50 μg of HI combined with 25 μg of c-di-AMP or 50 μL of AddaVax, CFA or AlPO_4_ in a total volume of 100 μL with adjuvant or PBS alone on days 0, 14, and 21. Incomplete Freund’s Adjuvant (IFA) was used as adjuvant for boost immunization in CFA group. All the adjuvants used in this study were purchased from InvivoGen (San Diego, CA, USA).To determine the protective efficacies of the immunodominant epitopes, mice (n=10) were intramuscularly immunized with 75 μg of each epitope-KLH conjugate combined with 50 μL of AlPO_4_ on days 0, 14, and 21. One week after the last booster, mice were infected with 9×10^8^ CFU of *S. aureus* strain MRSA252 (ATCC, Manassas, VA, USA) in 100 μL saline, and the survival rates in each group were monitored for 7 days following infection.

### Bacterial Burden and Tissue Histology

Mice in each group were infected with 6×10^8^ CFU of MRSA252 7 days after the last immunization (n=8), and kidneys and lungs were harvested 48 h post infection. The bacterial burden in the organs was quantified by preparing organ homogenates in PBS and plating 10-fold serial dilutions on MHA plates. The colonies were counted after 24 h of incubation at 37°C. The number of CFU per tissue (CFU/organ) was calculated from each plate. For histopathology, the organs were fixed in 4% paraformaldehyde and embedded in paraffin. Four-micrometer thick sections were prepared and stained with hematoxylin and eosin for microscopic examination. Each lung section of PBS- and HI-immunized mice was given a score of 0–4 (no abnormality to most severe) according to established criteria based on hyperemia, edema, hemorrhage, and neutrophil infiltration.

### Immunoglobulin Subtyping

One week after the last immunization, mice were exsanguinated, and serum samples were collected. The titers of HI-specific IgG were determined by ELISA. In brief, wells of microtiter plates (Thermo Lab Systems) were coated with HI (5 µg per well) in 50 mM carbonate buffer (pH9.5) at 4°C overnight. Nonspecific binding was blocked with 2% BSA (v/v) at 37°C for 2 h. Serum samples were serially diluted 2-fold in PBS (starting at 1:1000) and used as the primary antibodies. The secondary antibodies were HRP-conjugated goat anti-mouse IgG (Sigma). Absorbance was read at 450 nm (OD450), and the titers were defined as the highest dilution that yielded an absorbance value of more than twice the value of the pre-immune serum. To determine the subtype of HI-specific IgG, serum samples diluted at 1:1000 were used as the primary antibodies, and HRP-conjugated goat anti-mouse IgG1, IgG2a, IgG2b and IgG3 (Sigma) were used as secondary antibodies.

### Cytokine Assays

One week after the last immunization, mice were infected with 6×10^8^ CFU of MRSA252. Two days after infection, each mouse was euthanized with CO_2,_ and serum was harvested using a 1-mL syringe. Samples were centrifuged at 8,000 rpm for 5 min, and the supernatants were collected and stored at -80°C until analysis. Cytokine levels in the serum were determined using the Cytometric Bead Array-based FlowCytomix assay according to the manufacturer’s instructions (LEGENDplex™ Panel, Biolegend, Cat No. 740446) for the following proinflammatory cytokines: interleukin (IL)-1α, IL-6, MCP-1, IL-17A, tumor necrosis factor (TNF)-α, and interferon (IFN)-γ. Samples were allowed to thaw at room temperature only once prior to testing. All assays were performed in duplicate.

### Linear B-Cell Epitope Mapping

To determine the reactivity of serum samples from immunized mice against each peptide, wells of microtiter plates were coated with 5 µM of each peptide dissolved in 50 mM carbonate buffer (pH9.6), and OVA_192–201_ was used as a negative control peptide. Nonspecific binding was prevented by blocking the coated microtiter plates with phosphate buffered saline (PBS, pH7.4) containing 2% BSA. Serum samples from immunized mice (n=10) were diluted in PBS at a ratio of 1:300 (v/v) and were used as the primary antibodies, and peroxidase-conjugated goat anti-mouse IgG antibodies (Solarbio, Beijing, China) were used as secondary antibodies at a dilution of 1:3500. The ELISA results are shown as absorbance values observed at 450 nm. The normal value for each peptide was calculated by testing sera from healthy mouse sera without immunization, and values that were 2.1-fold higher than the mean absorbance value of negative sera were defined as positive.

### Structural Localization and Sequence Alignment of the Immunodominant Epitopes

The crystal structures of both Hla (3anz.pdb) (17) and IsdB (3rtl.pdb) (18) were obtained from the protein database (PDB). Immunodominant epitopes were located on these structures using the PyMOL 1.1 program. Sequences of Hla and IsdB from different *S. aureus* strains were retrieved from the GenBank database for alignment using the NCBI Basic Local Alignment Search Tool (BLAST) software.

### Statistical Analysis

Statistical analyses were performed using GraphPad Prism 8.0. All data are presented as the mean ± standard deviation (S.D.). Data were analyzed using one-way ANOVA with Bonferroni correction. Survival data were analyzed using the log-rank (Mantel-Cox) test. P < 0.01 was considered statistically significant.

## Results

### Immunization With HI Formulated With Different Adjuvants Provides Different Protective Efficacies Against MRSA252 Challenge

**Figure 1:**
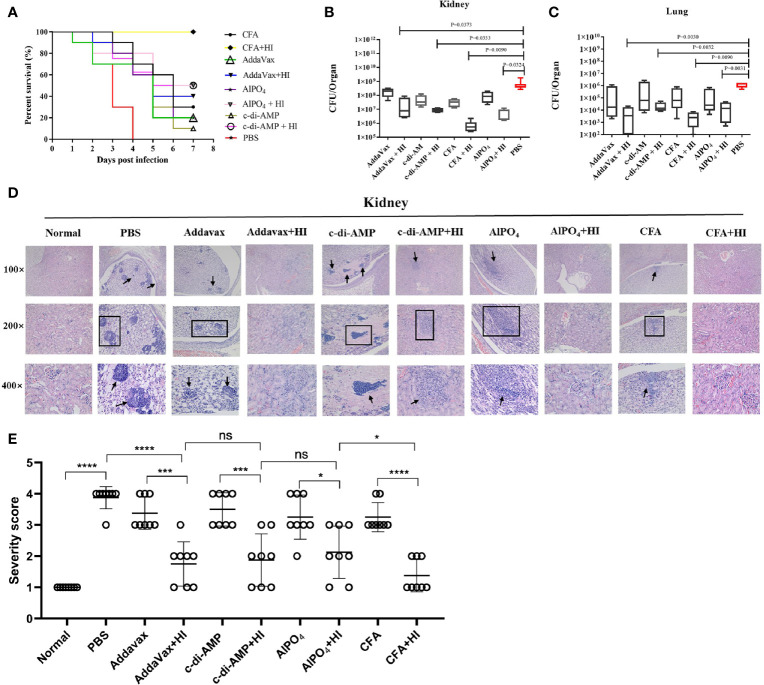
The protective efficacy of immunization with HI formulated with different adjuvants against MRSA252 challenge. **(A)** Percent survival in mice immunized with HI plus CFA, AlPO_4_, AddaVax or c-di-AMP adjuvant, adjuvant alone or PBS as a control (n =10). Bacterial burden in the kidneys **(B)** and lungs **(C)** of immunized mice after challenge with MRSA252 (n = 8). The difference in each group is indicated by the p value. **(D)** Histological analysis of immunized mice after challenge with MRSA252. Hematoxylin and eosin staining of kidney sections 48 h after MRSA252 infection, 100 × (top row), 200 × (middle row) and 400 × (bottom row). Abscess formation or scattered colonies of bacteria were found only in adjuvants alone or PBS-immunized animals (black arrow). **(E)** Severity scores of lungs (n = 8) from immunized mice and control mice are shown 2 days postinfection. Data are presented as scatter plots. ns, not significant; *p < 0.05, ***p < 0.001, ****p < 0.0001.

BALB/c mice were immunized with HI plus different adjuvants on days 0, 14 and 21 and then challenged with a lethal dose of MRSA252 one week after the last immunization. As shown in **Figure 1A**, all mice in the PBS-immunized group died within 4 days after MRSA252 infection, consistent with our previous studies and indicating that the bacteremia model was successful (13). In contrast, mice immunized with HI and different adjuvants exhibited different protective efficacies. The highest survival rate was observed in the HI plus CFA immunized group, which showed 100% survival without any clinical symptoms after MRSA252 challenge. The survival rate in this group was significantly higher than that in the other groups. Moreover, HI immunized with AlPO_4_ and c-di-AMP showed the same survival rate (50%), which was slightly higher than that of mice immunized with HI plus AddaVax (40%). The significance of protective immunity generated by the HI and different adjuvants was measured using the log-rank(Mantel-Cox)test compared to the PBS group, CFA plus HI (P<0.0001), AlPO_4_ plus HI (P= 0.0787), c-di-AMP plus HI (P = 0.1832), AddaVax plus HI (P = 0.2910), CFA alone (P = 0.2695), AlPO_4_ alone (P = 0.8943), c-di-AMP alone (P = 0.5932), and AddaVax alone (P = 0.9114). These results illustrated that immunization with HI plus different adjuvants resulted in different protective efficacies for controlling MRSA252 infection. Furthermore, mice immunized with each adjuvant alone also exhibited partial protection, but the survival rate was significantly lower than that of the HI immunization group, further demonstrating that HI is an effective antigen for the *S. aureus* vaccine (**Figure 1A**).

### Immunization With HI Plus Different Adjuvants Reduces MRSA252 Infection in a Bacteremia Model

The organs of mice immunized with HI plus different adjuvants were assessed for bacterial load after challenge with a sub-lethal dose of MRSA252 *via* intravenous injection. As expected, HI plus different adjuvant-immunized mice resulted in significantly lower bacterial burden in kidney (**Figure 1B**) and lung tissues (**Figure 1C**) than in PBS-control counterparts and adjuvant-immunized mice. Notably, the bacterial burden in mice immunized with HI plus CFA exhibited the lowest bacterial burden, both in kidney and lung tissues, and the bacterial burden in each group was negatively correlated with the survival rate.

Histological analysis of MRSA-challenged, HI plus different adjuvant-immunized mice revealed normal physiological architecture of the renal tubules and pulmonary alveoli with no detectable bacterial colonies, while adjuvant-immunized mice exhibited moderate changes in the physiological architecture of pulmonary alveoli (fibrin and serous effusion) and renal tubules (neutrophils). In comparison, scattered bacterial colonies were readily observed in the kidneys and lungs of the PBS control mice, particularly in the kidney abscesses (**Figure 1D**). The severe score of kidney tissue was consistent with that of bacterial burden and survival rate (**Figure 1E**). Taken together, these results indicate that HI plus different adjuvant immunizations provided more robust protection against MRSA252 challenge than that conveyed by PBS-control or adjuvant immunization alone.

### Host Immune Response Toward HI Immunization With Different Adjuvants

**Figure 2:**
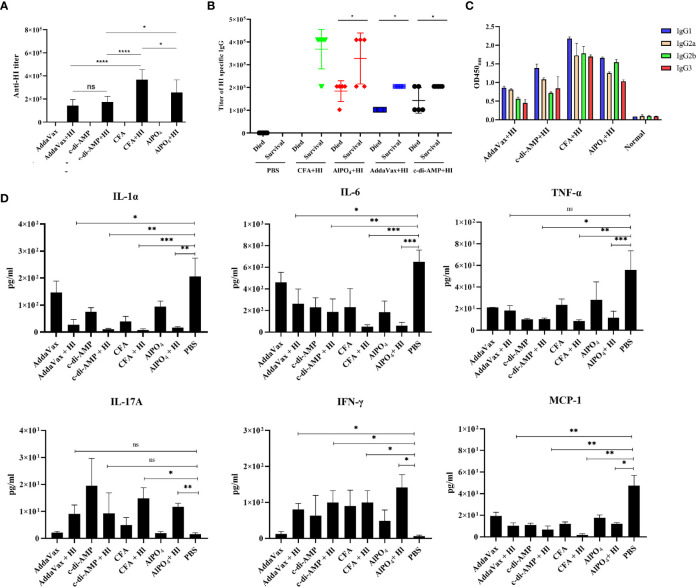
HI-specific antibody and cytokine production in response to MRSA252 challenge. **(A)** Antibody production in mice immunized with HI plus different adjuvants, adjuvants alone, or PBS alone. **(B)** Correlation between the titer of HI-specific IgG and the survival of each immunized mouse (n=10). **(C)** HI-specific antibody isotope analysis in mice immunized with HI plus different adjuvants, adjuvants alone, or PBS alone. Results are representative of three experiments, and data are shown as the mean ± SD of samples from 10 mice. **(D)** Cytokine analysis in the sera of immunized mice after challenge with MRSA252. Serum samples were collected 48 h after infection (n = 8). ns, not significant; *p < 0.05, **p < 0.01, ***p < 0.001, ****p < 0.0001.

As HI-mediated protection was primarily dependent on the humoral immune response (10), we next tested the titers and subtypes of HI-specific IgG in the sera of the immunized mice 7 days after the last booster immunization. As shown in **Figure 2A**, elevated levels of HI-specific IgG were detected in all mice immunized with HI plus adjuvants. Notably, the highest titer of IgG was observed in mice immunized with HI plus CFA, which was significantly higher than all the other groups. The titer of HI-specific IgG in mice immunized with HI plus AlPO_4_ was lower than in mice immunized with HI plus CFA (p<0.05) but significantly higher than the titers of mice immunized with HI plus two other adjuvants. Meanwhile, the titers of IgG in mice immunized with HI plus AddaVax and mice immunized HI plus c-di-AMP group were not significantly different. In contrast, the titers of HI-specific IgG from mice that survived MRSA252 infection were significantly higher than those from mice that died (**Figure 2B**). Serum IgG subtype analysis in mice immunized with HI plus different adjuvants revealed a primarily IgG1 response. The levels of subtypes showed a similar tendency to the titers of total IgG in each group, and there was no significant difference in the distribution of subtypes among the different groups (**Figure 2C**).

Levels of the cytokines IL-1α, IL-6, MCP-1, TNF-α, IL-17 and IFN-γ in serum were analyzed to evaluate the inflammatory response in immunized mice in response to MRSA252 challenge. As shown in **Figure 2D**, proinflammatory cytokines, such as IL-1α, IL-6 and TNF-α, were significantly decreased in HI-immunized mice compared to PBS-immunized mice (p<0.01). In contrast, levels of IFN-γ and IL-17A in the serum of immunized mice were increased after bacterial infection, suggesting that immunization with HI was capable of inducing IFN-γ- and IL-17A-producing cells and that cellular immunity might also be involved in HI-mediated protection against *S. aureus* infection. These results are consistent with previous studies reporting that cellular immunity plays essential roles in protective immunity induced by other *S. aureus* antigens, such as MntC or PBP2a (19, 20). Furthermore, MCP-1 is one of the key chemokines that regulates the migration and infiltration of monocytes/macrophages (21), and levels of MCP-1 in each group were similar to those of proinflammatory cytokines, indicating that inflammation in the lungs of HI-immunized mice was decreased compared to that in PBS-immunized mice.

### Identification of Immunodominant Epitopes on Hla and IsdB Using Serum From Mice Immunized With HI Plus Different Adjuvants

**Figure 3:**
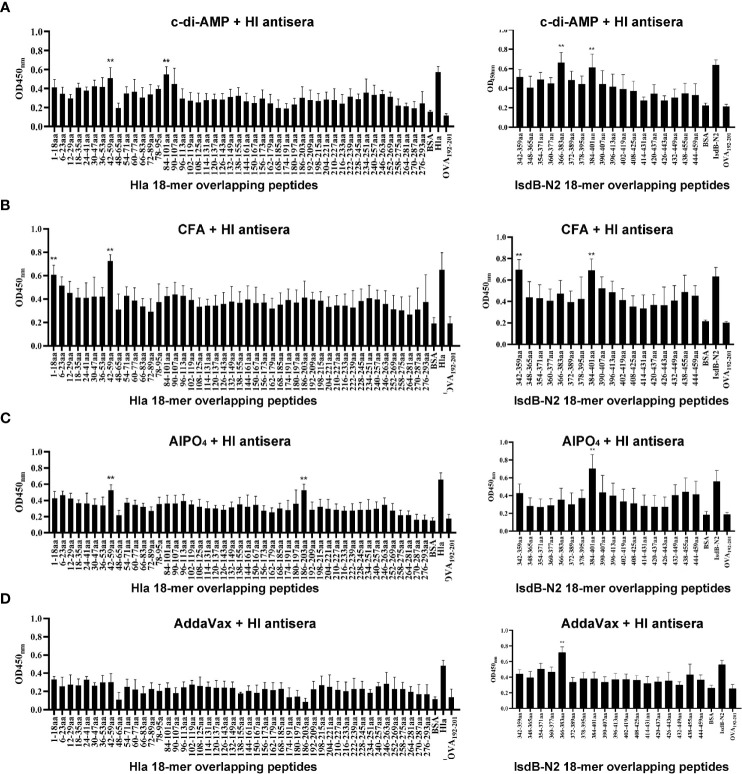
B cell epitope mapping in Hla or IsdB by ELISA. To identify the immunodominant epitopes on Hla or IsdB, microtiter plates were coated with synthetic overlapping peptides that spanned the entire length of the Hla or IsdB. OVA_192–201_ was used as a negative control peptide. Then, serum samples from BALB/c mice immunized with HI plus c-di-AMP **(A)**, CFA **(B)**, AlPO_4_ **(C)** and AddaVax **(D)** adjuvants were used as primary antibodies (n=10). The absorbance was read at 450 nm. The raw O.D. values shown were obtained using serum from three independent experiments assayed concurrently. Each bar represents the average OD observed for all 10 mice in each group. Data are represented as the means ± SEM. Probability values of p < 0.01 were considered significant and are denoted by an asterisk (**).

Linear B-cell epitope mapping was performed by ELISA using the overlapping 18-mer peptides and antiserum from mice immunized with HI and different adjuvants. As shown in **Figure 3**, seven epitopes (Hla_1-18_, Hla_42-59_, Hla_84-101_, Hla_186-203_, IsdB_342-359_, IsdB_366-383_ and IsdB_384-401_) were identified with strong IgG reactivity, and the sequences of these epitopes are listed in **Table 1**. Among these immunodominant epitopes, both Hla_42-59_ and IsdB_384-401_ were immunodominant in the HI plus c-di-AMP, AlPO4 and CFA groups. IsdB_366-383_ was immunodominant in the HI plus AddaVax and c-di-AMP groups, whereas the other four epitopes were only immunodominant in one group. These results suggested that adjuvants are capable of driving the immunodominant response toward the same antigen, resulting in differences in protective efficacy. Meanwhile, the immunodominant epitope Hla_42-59_ identified in this study may share the same B cell epitope in Hla_48-65_, which was previously identified using serum from humans (13). The other six epitopes have not been previously reported and therefore might harbor novel linear B-cell epitopes.

**Table 1:** Sequence of the immunodominant epitopes on Hla and IsdB identified in this study.

The immunodominant epitopes	Sequence of the immunodominant epitopes	The immunization group
Hla_1-18_	ADSDINIKTGTTDIGSNT	CFA+HI
Hla_42-59_	FIDDKNHNKKILVIRTKG	c-di-AMP+HI, AlPO_4_+HI, CFA+HI
Hla_84-101_	FKVQLQLPDNEVAQISDY	c-di-AMP+HI
Hla_186-203_	SWNPVYGNQLFMKTRNGS	AlPO_4_+HI
IsdB_342-359_	KMTDLQDTKYVVYESVEN	CFA+HI
IsdB_366-383_	AFVKHPIKTGMLNGKKYM	AddaVax+HI, c-di-AMP+HI
IsdB_384-401_	VMETTNDDYWKDFMVEGQ	CFA+HI, AlPO_4_+HI, c-di-AMP+HI

Next, we determined the protective efficacy of each single immunodominant epitope against MRSA252 infection. Mice were immunized three times with epitope-KLH conjugations and subsequently infected with a lethal dose of MRSA252 as described previously. As shown in **Supplementary Figure 1**, results demonstrated that all of these peptides provided partial protection against MRSA252 infection, ranging from 10% to 30%, when compared to the negative control. However, only Hla_1-18_-, Hla_42-59_- and IsdB_366-383_-KLH immunized groups showed differences in survival when compared to PBS immunized group, and no significant differences were observed among these epitope immunized groups compared to adjuvant immunized group, which is understandable since a single immunodominant epitope was unable to induce robust protection.

### Localization and Sequence Alignment of the Immunodominant Epitopes on Hla and IsdB

**Figure 4:**
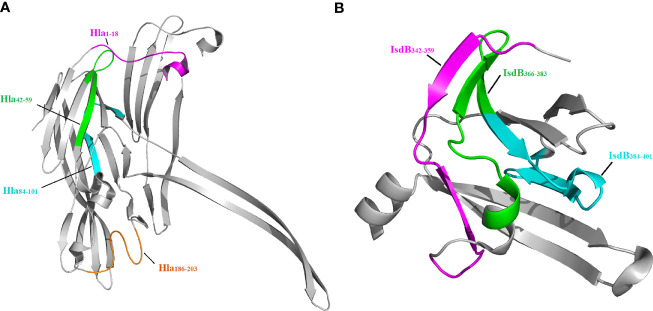
Localization of immunodominant epitopes on Hla and IsdB. The crystal structures of Hla (3anz.pdb) and IsdB (3rtl.pdb) were obtained from PDB. Immunodominant epitopes of Hla **(A)** and IsdB **(B)** were located on these structures using the PyMOL 1.1 program..

Since the crystal structures of Hla and IsdB are available in the PDB, we next located the seven epitopes on the structure of these proteins. As shown in **Figure 4**, all seven epitopes are located on the surface of these structures, making them more accessible to specific antibodies. Among these epitopes, Hla_42-59_, Hla_84-101_, IsdB_342-359_, IsdB_366-383_ and IsdB_384-401_ fold into a β-sheet structure. In contrast, the other two epitopes, Hla_1-18_ and Hla_186-203_, assemble into a complex of loop and α-helix structures.

**Figure 5:**
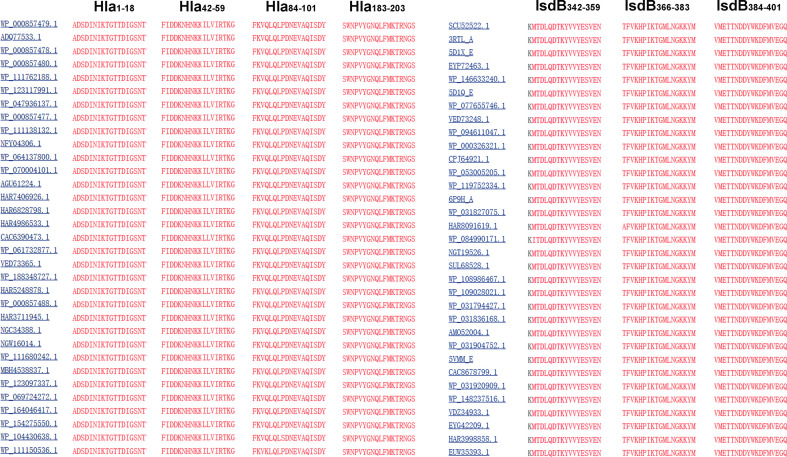
Sequence alignment of the immunodominant epitopes on Hla and IsdB. The sequences of Hla and IsdB from 33 different *S. aureus* strains were retrieved from the GenBank database. These sequences were aligned using the NCBI Basic Local Alignment Search Tool (BLAST) software.

To determine the conservation of these immunodominant epitopes, the amino acid sequences of Hla and IsdB from 33 randomly selected *S. aureus* strains were retrieved from the GenBank database for alignment. The results revealed that sequences of all seven immunodominant epitopes identified in this study were completely conserved among these *S. aureus* strains, with amino acid identities of 100% (**Figure 5**). As a result, specific antibodies targeting these epitopes may cross-react with different strains of *S. aureus*.

## Discussion

Vaccination has proven to be the most cost-effective strategy to combat infectious diseases. Due to the high morbidity and mortality associated with *S. aureus* infections and the steady increase in the frequency of drug-resistant isolates, there is an urgent need to develop vaccines against these bacteria (22). Our team developed the pentavalent *S. aureus* vaccine rFSAV, the average protection rate of which was 87% in an animal model (9). Furthermore, a phase I clinical trial demonstrated that rFSAV was immunogenic and well tolerated in healthy volunteers and that both Hla and IsdB induced a robust and specific immune response (to be published). Hla is a pathogenic exotoxin with hemolytic activity secreted by *S. aureus* that plays an important role in bacteremia caused by *S. aureus* infection (23, 24). Previous reports have shown that antibody levels of Hla are positively correlated with the survival of patients with *S. aureus* bacteremia (25), and humanized Hla monoclonal antibodies can effectively eradicate *S. aureus* infection in pneumonia patients in the ICU (26). IsdB is distributed on the surface of *S. aureus* and acts as a receptor for hemoglobin (18), which functions to maintain the growth of *S. aureus* and is also closely related to the pathogenesis of endocarditis caused by *S. aureus* infection (27).

Although antigens are the most important components within a vaccine, the vaccination strategy and the co-administration of adjuvants are also crucial for the protective immune response (28). Therefore, the precise monitoring of adjuvant candidates is an essential step for vaccine design. In the current study, we determined the humoral immune response and protective efficacies of HI formulated using four different adjuvants. Among them, CFA consists of heat-inactivated *Mycobacterium tuberculosis* in nonmetabolizable oils. This adjuvant is widely used in animal experiments but has not yet been approved for human use and is considered to be the most effective adjuvant available for consistently producing high titer antibodies to diverse antigens (29). AlPO_4_ is a classic adjuvant that is used in most approved vaccines. It enhances the immune response by facilitating phagocytosis and slowing the diffusion of antigens from the site of injection, and it efficiently induces a Th2 immune response (30). AddaVax is a squalene-based oil-in-water nanoemulsion that efficiently elicits both cellular and humoral immune responses (31). The formulation of AddaVax is similar to that of MF59, which is already used in flu vaccines (32). c-di-AMP is a STING activator that promotes high humoral and cellular immune responses to model and disease-related antigens and vaccines (33). All adjuvants used in this study proved to be efficient in inducing humoral immunity against HI.

The protective response induced by antigens is essentially and is primarily the result of specific protective immunodominant epitopes; therefore, epitope mapping is critical for understanding the protective mechanism of an antigen. The immune responses induced by different immunodominant epitopes of the same antigen, as well as the different levels of immune responses elicited by the same immunodominant epitope, can both result in different protective efficacies. In this study, we found that mice immunized with HI from *S. aureus* plus different adjuvants exhibited a significant difference in response to *S. aureus* infection in a bacteremia model, and survival rates of the immunization groups varied from 40% to 100%. As expected, the titers of HI-specific antibodies were correlated with survival rates, consistent with previous findings that the humoral immune response is essential for HI-mediated protection (10).

In this study, mice were immunized with HI formulated with different adjuvants, and the titers of HI-specific IgG, IgG subtype, B cell linear epitope and the levels of inflammatory cytokines in immunized mice were further analyzed. The titers of HI-specific IgG were closely correlated with the survival rates in each group, and serum IgG subtype analysis in mice immunized with HI plus different adjuvants revealed a primarily IgG1 response. There was no significant difference in the distribution of subtypes among the different groups. In contrast, B cell epitope mapping revealed different immunodominant epitopes, suggesting that the difference in protective immunity observed in different immunization groups might be due, at least in part, to the different antibody titers induced in response to different adjuvant-mediated epitope specificities. As there were several immunodominant epitopes rather than a single immunodominant epitope in every immunization group, the immunodominant epitopes in the same immunization group play an additive or synergistic effect in protection against MRSA252 infection, which has been confirmed by several previous studies (34, 35). Sequence alignment indicated that all of these epitopes were conserved among different isolates of *S. aureus*, indicating that antibodies recognizing these epitopes might provide broad protection against infections caused by different *S. aureus* isolates.

Cytokine assays revealed that levels of IL-1α, IL-6, TNF-α and MCP-1 in the serum of HI-immunized mice were lower than those in the serum of control mice, suggesting that HI immunization reduced levels of inflammation in response to *S. aureus* infection. Meanwhile, levels of IFN-γ and IL-17A were higher in HI-immunized mice than in PBS-immunized mice, indicating that the cellular immune response might also be involved in HI-mediated protection. Similar results have been reported by former studies. For example, one report indicated that IFN-γ plays an essential role in murine defense against *S. aureus* nasal infection (36). Another study reported that immunization with the fibrinogen-binding domain of clumping factor A, a candidate antigen from *S. aureus*, efficiently induced IL-17A-producing cells and showed that IL-17-mediated cellular immunity was involved in the protective effect against *S. aureus* infection (37).

In summary, we identified seven B cell epitopes in the HI antigen using serum from mice immunized with HI plus different adjuvants. This study may shed light on the molecular mechanisms of Hla and IsdB-mediated protection against *S. aureus* infection. It is very important to select the appropriate adjuvant to optimize the protective efficacy of rFSAV since the adjuvants AlPO_4_, CFA, AddaVax and c-di-AMP plus the same antigen HI induced different immunodominant B cell responses. Further studies will be needed to determine whether the differential protection levels obtained with each adjuvant are due to differences in antibody titers, immunodominant epitopes, cellular responses, or a combination of all these factors.

## Data Availability Statement

The original contributions presented in the study are included in the article/**Supplementary Material**. Further inquiries can be directed to the corresponding authors.

## Ethics Statement 

The animal study was reviewed and approved by The Animal Ethical and Experimental Committee of the Third Military Medical University (Chongqing, Permit No. 2011-04).

## Author Contributions

JYZ, ZZ, HZ, and QZ designed research. ZC, QG, QX, LD, HJ, and YY performed the experiments. JYZ, ZZ, and XZ analyzed the data. JTZ, LC, and PL contributed reagents/materials/analysis tools. ZZ and JYZ wrote the paper. All authors contributed to the article and approved the submitted version.

## Conflict of Interest

The authors declare that the research was conducted in the absence of any commercial or financial relationships that could be construed as a potential conflict of interest.

## Supplementary Material

Supplementary Figure 1 The protective efficacy of immunization with each single immunodominant epitope-KLH formulated with AlPO_4_ against MRSA252 challenge. Percent survival in mice immunized with every single immunodominant epitope-KLH plus AlPO_4_ adjuvant, AlPO_4_ alone or PBS was used as control (n =10). *p < 0.05, **p < 0.01, compared with PBS group.

## References

[1] Govindaraj Vaithinathan A Vanitha A. WHO Global Priority Pathogens List on Antibiotic Resistance: An Urgent Need for Action to Integrate One Health Data. Perspect Public Health (2018) 138:87–8. 10.1177/1757913917743881 29465015

[2] Ortwine JK Bhavan K. Morbidity, Mortality, and Management of Methicillin-Resistant S. Aureus Bacteremia in the USA: Update on Antibacterial Choices and Understanding. Hosp Practice (2018) 46:64–72. 10.1080/21548331.2018.1435128 29400119

[3] Tong SY Davis JS Eichenberger E Holland TL Fowler VG Jr. Staphylococcus Aureus Infections: Epidemiology, Pathophysiology, Clinical Manifestations, and Management. Clin Microbiol Rev (2015) 28:603–61. 10.1128/CMR.00134-14 PMC4451395 26016486

[4] Fattom A Matalon A Buerkert J Taylor K Damaso S Boutriau D. Efficacy Profile of a Bivalent Staphylococcus Aureus Glycoconjugated Vaccine in Adults on Hemodialysis: Phase III Randomized Study. Hum Vaccines Immunotherapeutics (2015) 11:632–41. 10.4161/hv.34414 PMC4514248 25483694

[5] Frenck RW Jr. Creech CB Sheldon EA Seiden DJ Kankam MK Baber J. Safety, Tolerability, and Immunogenicity of a 4-Antigen Staphylococcus Aureus Vaccine (SA4Ag): Results From a First-in-Human Randomised, Placebo-Controlled Phase 1/2 Study. Vaccine (2017) 35:375–84. 10.1016/j.vaccine.2016.11.010 27916408

[6] Harro CD Betts RF Hartzel JS Onorato MT Lipka J Smugar SS. The Immunogenicity and Safety of Different Formulations of a Novel Staphylococcus Aureus Vaccine (V710): Results of Two Phase I Studies. Vaccine (2012) 30:1729–36. 10.1016/j.vaccine.2011.12.045 22192849

[7] Fattom AI Horwith G Fuller S Propst M Naso R. Development of StaphVAX, a Polysaccharide Conjugate Vaccine Against S. Aureus Infection: From the Lab Bench to Phase III Clinical Trials. Vaccine. (2004) 22:880–7. 10.1016/j.vaccine.2003.11.034 15040941

[8] Redi D Raffaelli CS Rossetti B De Luca A Montagnani F. Staphylococcus Aureus Vaccine Preclinical and Clinical Development: Current State of the Art. New Microbiol (2018) 41:208–13. 29874390

[9] Zeng H Yang F Feng Q Zhang J Gu J Jing H. Rapid and Broad Immune Efficacy of a Recombinant Five-Antigen Vaccine Against Staphylococcus Aureus Infection in Animal Models. Vaccines (2020) 8(1):134. 10.3390/vaccines8010134 PMC7157245 32197534

[10] Zuo QF Yang LY Feng Q Lu DS Dong YD Cai CZ. Evaluation of the Protective Immunity of a Novel Subunit Fusion Vaccine in a Murine Model of Systemic MRSA Infection. PloS One (2013) 8:e81212. 10.1371/journal.pone.0081212 24324681 PMC3852261

[11] Zhang J Yang F Zhang X Jing H Ren C Cai C. Protective Efficacy and Mechanism of Passive Immunization With Polyclonal Antibodies in a Sepsis Model of Staphylococcus Aureus Infection. Sci Rep (2015) 5:15553. 10.1038/srep15553 26490505 PMC4614693

[12] Aw-Yong KL Sam IC Koh MT Chan YF. Immunodominant IgM and IgG Epitopes Recognized by Antibodies Induced in Enterovirus A71-Associated Hand, Foot and Mouth Disease Patients. PloS One (2016) 11:e0165659. 10.1371/journal.pone.0165659 27806091 PMC5091889

[13] Zhao Z Song X Zeng J Yuan Y Chen Z Xu L. An Immunodominant Epitope-Specific Monoclonal Antibody Cocktail Improves Survival in a Mouse Model of Staphylococcus Aureus Bacteremia. J Infect Dis (2020). 10.1093/infdis/jiaa602 32959055

[14] Del Giudice G Rappuoli R Didierlaurent AM. Correlates of Adjuvanticity: A Review on Adjuvants in Licensed Vaccines. Semin Immunol (2018) 39:14–21. 10.1016/j.smim.2018.05.001 29801750

[15] Awate S Babiuk LA Mutwiri G. Mechanisms of Action of Adjuvants. Front Immunol (2013) 4:114. 10.3389/fimmu.2013.00114 23720661 PMC3655441

[16] Wang X Tai W Zhang X Zhou Y Du L Shen C. Effects of Adjuvants on the Immunogenicity and Efficacy of a Zika Virus Envelope Domain III Subunit Vaccine. Vaccines (2019) 7(4):161. 10.3390/vaccines7040161 PMC6963592 31717890

[17] Tanaka Y Hirano N Kaneko J Kamio Y Yao M Tanaka I. 2-Methyl-2,4-pentanediol Induces Spontaneous Assembly of Staphylococcal Alpha-Hemolysin Into Heptameric Pore Structure. Protein Sci: Publ Protein Society (2011) 20:448–56. 10.1002/pro.579 PMC3048429 21280135

[18] Gaudin CF Grigg JC Arrieta AL Murphy ME. Unique Heme-Iron Coordination by the Hemoglobin Receptor IsdB of Staphylococcus Aureus. Biochemistry (2011) 50:5443–52. 10.1021/bi200369p PMC3114464 21574663

[19] Mortazavi SS Haghighat S Mahdavi M. Recombinant PBP2a of Methicillin-Resistant S. Aureus Formulation in Alum and Montanide ISA266 Adjuvants Induced Cellular and Humoral Immune Responses With Protection in Balb/C Mice. Microb Pathogeb (2020) 140:103945. 10.1016/j.micpath.2019.103945 31874228

[20] Yu W Yao D Yu S Wang X Li X Wang M. Protective Humoral and CD4(+) T Cellular Immune Responses of Staphylococcus Aureus Vaccine MntC in a Murine Peritonitis Model. Sci Rep (2018) 8:3580. 10.1038/s41598-018-22044-y 29483570 PMC5832154

[21] Deshmane SL Kremlev S Amini S Sawaya BE. Monocyte Chemoattractant Protein-1 (MCP-1): An Overview. J Interferon Cytokine Res (2009) 29:313–26. 10.1089/jir.2008.0027 PMC2755091 19441883

[22] Kourtis AP Hatfield K Baggs J Mu Y See I Epson E. Vital Signs: Epidemiology and Recent Trends in Methicillin-Resistant and in Methicillin-Susceptible Staphylococcus Aureus Bloodstream Infections - United States. MMWR Morbidity Mortality Weekly Rep (2019) 68:214–9. 10.15585/mmwr.mm6809e1 PMC6421967 30845118

[23] Kebaier C Chamberland RR Allen IC Gao X Broglie PM Hall JD. Staphylococcus Aureus Alpha-Hemolysin Mediates Virulence in a Murine Model of Severe Pneumonia Through Activation of the NLRP3 Inflammasome. J Infect Diseases (2012) 205:807–17. 10.1093/infdis/jir846 PMC3274379 22279123

[24] Rauch S DeDent AC Kim HK Bubeck Wardenburg J Missiakas DM Schneewind O. Abscess Formation and Alpha-Hemolysin Induced Toxicity in a Mouse Model of Staphylococcus Aureus Peritoneal Infection. Infection Immunity (2012) 80:3721–32. 10.1128/IAI.00442-12 PMC3457571 22802349

[25] Sharma-Kuinkel BK Wu Y Tabor DE Mok H Sellman BR Jenkins A. Characterization of Alpha-Toxin Hla Gene Variants, Alpha-Toxin Expression Levels, and Levels of Antibody to Alpha-Toxin in Hemodialysis and Postsurgical Patients With Staphylococcus Aureus Bacteremia. J Clin Microbiol (2015) 53:227–36. 10.1128/JCM.02023-14 PMC4290928 25392350

[26] Francois B Mercier E Gonzalez C Asehnoune K Nseir S Fiancette M. Safety and Tolerability of a Single Administration of AR-301, A Human Monoclonal Antibody, in ICU Patients With Severe Pneumonia Caused by Staphylococcus Aureus: First-in-Human Trial. Intensive Care Med (2018) 44:1787–96. 10.1007/s00134-018-5229-2 30343314

[27] Zapotoczna M Jevnikar Z Miajlovic H Kos J Foster TJ. Iron-Regulated Surface Determinant B (IsdB) Promotes Staphylococcus Aureus Adherence to and Internalization by Non-Phagocytic Human Cells. Cell Microbiol (2013) 15:1026–41. 10.1111/cmi.12097 23279065

[28] Shi S Zhu H Xia X Liang Z Ma X Sun B. Vaccine Adjuvants: Understanding the Structure and Mechanism of Adjuvanticity. Vaccine (2019) 37:3167–78. 10.1016/j.vaccine.2019.04.055 31047671

[29] Portuondo DL Ferreira LS Urbaczek AC Batista-Duharte A Carlos IZ. Adjuvants and Delivery Systems for Antifungal Vaccines: Current State and Future Developments. Med Mycology (2015) 53:69–89. 10.1093/mmy/myu045 25362733

[30] Mei C Deshmukh S Cronin J Cong S Chapman D Lazaris N. Aluminum Phosphate Vaccine Adjuvant: Analysis of Composition and Size Using Off-Line and In-Line Tools. Comput Struct Biotechnol J (2019) 17:1184–94. 10.1016/j.csbj.2019.08.003 PMC6739432 31528298

[31] Choubini E Habibi M Khorshidi A Ghasemi A Asadi Karam MR Bouzari S. A Novel Multi-Peptide Subunit Vaccine Admixed With AddaVax Adjuvant Produces Significant Immunogenicity and Protection Against Proteus Mirabilis Urinary Tract Infection in Mice Model. Mol Immunol (2018) 96:88–97. 10.1016/j.molimm.2018.03.001 29525454

[32] O’Hagan DT Wack A Podda A. MF59 is a Safe and Potent Vaccine Adjuvant for Flu Vaccines in Humans: What Did We Learn During Its Development? Clin Pharmacol Ther (2007) 82:740–4. 10.1038/sj.clpt.6100402 17971820

[33] Volckmar J Knop L Stegemann-Koniszewski S Schulze K Ebensen T Guzman CA. The STING Activator c-di-AMP Exerts Superior Adjuvant Properties Than the Formulation Poly(I:C)/Cpg After Subcutaneous Vaccination With Soluble Protein Antigen or DEC-205-mediated Antigen Targeting to Dendritic Cells. Vaccine (2019) 37:4963–74. 10.1016/j.vaccine.2019.07.019 31320219

[34] Batista MT Ferreira EL Pereira GS Stafford P Maeda D Rodrigues JF. LT Adjuvant Modulates Epitope Specificity and Improves the Efficacy of Murine Antibodies Elicited by Sublingual Vaccination With the N-terminal Domain of Streptococcus Mutans P1. Vaccine (2017) 35:7273–82. 10.1016/j.vaccine.2017.11.007 29146379

[35] Maeda D Batista MT Pereira LR de Jesus Cintra M Amorim JH Mathias-Santos C. Adjuvant-Mediated Epitope Specificity and Enhanced Neutralizing Activity of Antibodies Targeting Dengue Virus Envelope Protein. Front Immunol (2017) 8:1175. 10.3389/fimmu.2017.01175 28993770 PMC5622152

[36] DeForge LE Billeci KL Kramer SM. Effect of IFN-gamma on the Killing of S. Aureus in Human Whole Blood. Assessment of Bacterial Viability by CFU Determination and by a New Method Using Alamarblue. J Immunological Methods (2000) 245:79–89. 10.1016/s0022-1759(00)00279-9 11042285

[37] Cho JS Pietras EM Garcia NC Ramos RI Farzam DM Monroe HR. Il-17 is Essential for Host Defense Against Cutaneous Staphylococcus Aureus Infection in Mice. J Clin Invest (2010) 120:1762–73. 10.1172/JCI40891 PMC2860944 20364087

