# Supplementary figures and images for: Immunodominance of Epitopes and Protective Efficacy of HI Antigen Are Differentially Altered Using Different Adjuvants in a Mouse Model of Staphylococcus aureus Bacteremia
Source: Front Immunol. 2021 May 27;12:684823. doi: 10.3389/fimmu.2021.684823 (PMC8190387; doi:10.3389/fimmu.2021.684823)

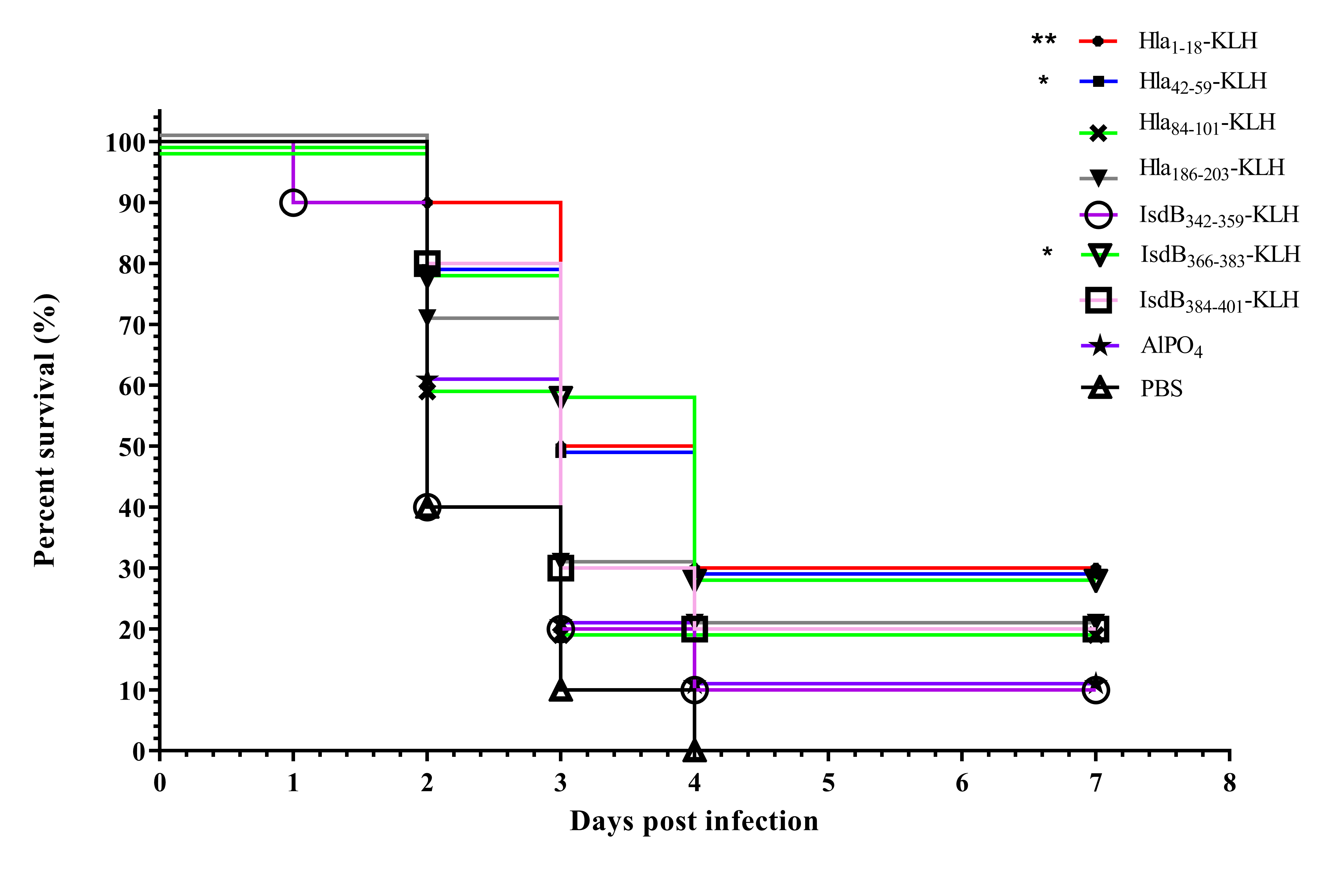

Supplement: Supplementary Figure 1 — The protective efficacy of immunization with each single immunodominant epitope-KLH formulated with AlPO4 against MRSA252 challenge. Percent survival in mice immunized with every single immunodominant epitope-KLH plus AlPO4 adjuvant, AlPO4 alone or PBS was used as control (n =10). *p < 0.05, **p < 0.01, compared with PBS group. [file Image_1.tif]
